# Overnight pulse wave analysis to assess autonomic changes during sleep in insomnia patients and healthy sleepers

**DOI:** 10.1371/journal.pone.0232589

**Published:** 2020-05-07

**Authors:** Naima Laharnar, Ludger Grote, Ding Zou, Jan Hedner, Dirk Sommermeyer, Christian Straßberger, Albert Marciniak, Sabrina Potzka, Katharina Lederer, Martin Glos, Sandra Zimmermann, Ingo Fietze, Thomas Penzel

**Affiliations:** 1 Interdisciplinary Center of Sleep Medicine, Charité –Universitätsmedizin Berlin, Berlin, Germany; 2 Center for Sleep and Vigilance Disorders, Institute of Medicine, Sahlgrenska Academy, University of Gothenburg, Gothenburg, Sweden; 3 Institute for Digital Signal Processing, Faculty of Information Technology, University Mannheim, Mannheim, Germany; 4 Advanced Sleep Research GmbH, Berlin, Germany; 5 Saratov State University, Saratov, Russia; University of Rome Tor Vergata, ITALY

## Abstract

Insomnia has been associated with increased cardiovascular (CV) risk, which may be linked to sympathetic activation. Non-invasive overnight pulse wave analysis may be a useful tool to detect early signs of autonomic changes during sleep in insomniacs. Fifty-two participants (26 men, 37±13 years, BMI: 24±5 kg/m^2^, 26 insomniacs/ 26 controls) underwent overnight polysomnography with pulse oximetry and pulse wave analysis including pulse rate, vascular stiffness (pulse propagation time, PPT), and a composite cardiac risk index based on autonomic function and overnight hypoxia. We identified two subgroups of insomniacs, with and without objectively disturbed sleep (sleep efficiency SE≤80%, n = 14 vs. SE>80%, n = 12), and observed increased pulse rate and vascular stiffness in insomnia cases when diagnosis was based on both, subjective and objective criteria. Both insomnia groups were associated with higher overnight pulse rate than controls (median/ IQR: low-SE (low sleep efficiency): 67/ 58-70bpm; high-SE: 66/ 63-69bpm; controls: 58/ 52-63bpm; *p = 0*.*01*). Vascular stiffness was higher (reduction of PPT) in low-SE insomniacs compared with high-SE insomniacs and controls (169/ 147-232ms; 237/ 215-254ms; 244/ 180-284ms; *p = 0*.*01*). The cardiac risk index was increased in low-SE insomniacs (0.2/ 0.0–0.7; 0.0/ 0.0–0.4; 0.0/ 0.0–0.3; *p = 0*.*05*). Our results suggest a hyperarousal state in young and otherwise healthy insomniacs during sleep. The increased pulse rate and vascular stiffness in insomniacs with low SE suggest early signs of rigid vessels and potentially, an elevated CV risk. Overnight pulse wave analysis may be feasible for CV risk assessment in insomniacs and may provide a useful tool for phenotyping insomnia in order to provide individualized therapy.

## Introduction

Cardiovascular (CV) diseases account for 30% of all deaths worldwide [1]. Risk assessment is relevant for effective treatment and reduction of mortality rates [2]. Conventional CV risk assessment including the ESC/ESH (European Society of Cardiology/ European Society of Hypertension) risk prognostic matrix is based on quantification of established risk factors (e.g., hypertension, obesity, age, sex, smoking, diabetes; [3]). However, the predictive accuracy of such components is limited, which is why imaging techniques, genetic tests, biomarkers and various functional assessment of the CV system (e.g., heart rate variability analysis, 24-h blood pressure assessment, pulse wave velocity measurement) have been applied to provide additional information [4,5].

Photoplethysmography by a pulse oximeter finger sensor is an established, non-invasive, and low-cost method to record microvascular blood volumetric changes and to provide information on autonomic activity and cardiovascular information. As sensors can be incorporated into a wristband, this method is more accessible than e.g., electrocardiographic recordings. A light source illuminates the tissue and a photodetector identifies variations in light intensity. The oximeter signal mirrors the wave-like motion of the blood movement as the backscattered light matches the changes in blood volume [6]. Pulse wave analysis may be used to assess vascular stiffness and may provide an alternative to well-established methods such as blood pressure measurement for CV risk prediction.

In a previous study, we developed an autonomic state indicator algorithm based on photoplethysmographic signal decomposition, which was validated with a sleep laboratory cohort. Sensitivity and specificity for detection of high- versus low-risk individuals according to ESC/ESH risk scores were 80% and 77%, respectively [7]. Following optimization, the accuracy was confirmed in a multicenter sleep laboratory cohort [4,8]. While pulse wave analysis during sleep has been analyzed predominantly in subjects with sleep disorders like a sleep-disordered breathing, the basic physiological phenomena of pulse wave attenuation due to sympathetic activity have been evaluated in robust physiological experiments both during daytime and during sleep [9].

Features derived from the pulse wave signal have been shown to reflect the autonomic nervous system activity during daytime [10,11]. Overnight pulse wave analysis during sleep has not been systematically investigated in healthy individuals or patients with sleep disorders. However, such studies are important as ineffective sleep quality may have negative cardiovascular consequences [12–14]. Short sleep duration is linked to adverse hemodynamic and metabolic outcomes [15–17]. Insomnia is a common sleep disorder associated with a state of psychological and physiological hyperarousal [18,19]. Patients have difficulties initiating and maintaining sleep, which results in disrupted and reduced sleep with impaired daytime functioning [20]. Physiological hyperarousal is linked to altered autonomic function, high metabolic rate and heart rate, and reduced heart rate variability [15,21–23]. These physiological components may be addressed in a pulse wave analysis.

Our study investigated nighttime cardiovascular and autonomic signals during a parallel recording using full polysomnography (PSG) and pulse oximetry in insomnia patients and matched controls. We hypothesized that cardiovascular and autonomic activity assessed in the overnight pulse wave analysis were increased in insomnia patients compared with controls.

## Materials and methods

### Participants and recruitment

Patients with insomnia were recruited prospectively from the Center for Sleep Medicine at the Charité –Universiätsmedizin Berlin (Germany). Healthy volunteers were asked for participation following an advertisement. Participants were 18 to 60 years and had signed informed consent. Insomnia patients had a diagnosis of chronic insomnia according to ICSD-3 (International Classification of Sleep Disorders, 3^rd^ edition) with a score equal to or above 8 on the Insomnia Severity Index (ISI) questionnaire [20,24]. The control group contained healthy sleepers with an ISI score below 8 and were matched for gender, age, BMI, alcohol, smoking, and apnea-hypopnea index with the insomnia group. Exclusion criteria were use of hypnotics or any psychotropic substance, other previously known diagnoses of sleep or sleep-related disorders (narcolepsy or idiopathic hypersomnia, parasomnia, restless legs syndrome, periodic leg movement disorder, obstructive sleep apnea, or circadian rhythm disorder), a diagnosed psychiatric disorder that might influence sleep, neurological disease (stroke, epilepsy or brain damage), any condition requiring medication with a beta-blocker, use of an antihistamine, alcohol or drug abuse, excessive caffeine or nicotine use. In total, 75 subjects (insomniacs and controls) were screened. Twenty-three subjects were excluded from the study due to withdrawal of consent (n = 1), meeting one or more of the exclusion criteria (n = 2), constituting a technical drop out (electroencephalography or oximetry signal failure, n = 16), or exhibiting invalid or less than two hours of sleep recording in the PSG (n = 4). The study protocol was reviewed and approved by the Institutional Ethics and Scientific Review Committee at the Charité –Universitätsmedizin Berlin, Germany (EA 1/320/14).

### Procedures and data collection

Clinical history was assessed, a physical examination and a laboratory-based PSG study including an overnight pulse oximetry signal were performed. A certified somnologist confirmed a diagnosis of a chronic insomnia based on the assessments stated above. All participants completed the ISI questionnaire and a total ISI score of 0–7 implied healthy sleep and a score of 8–28 indicated insomnia (8–14 = mild insomnia, 15–21 = moderate insomnia, 22–28 = severe insomnia) [24]. Additionally, participants completed the Epworth Sleepiness Scale (ESS, a score > 10 indicated clinically significant sleepiness), the Pittsburgh Sleep Quality Index (PSQI, a score > 4 indicated clinically significant poor sleep quality), and the Restless Legs Syndrome–Diagnostic Index (RLS-DI, a score > 3 indicated a possible presence of RLS) [25–27]. The overnight PSG was conducted with the *SOMNOscreenplus* (Somnomedics, Randesacker, Germany) system, a portable full PSG system with wireless real-time data transmission, complying with AASM (American Academy of Sleep Medicine) criteria. Sensor placement was performed by trained sleep technicians in compliance with 2012 AASM rules [28]. The system recorded electroencephalography, electrocardiography, electrooculography, electromyography, nasal and oral flow, thoracic and abdominal efforts, body position, snoring, and pulse oximetry. In addition, the *SOMNOcheck micro CARDIO* (Weinmann Medical Technology GmbH + Co. KG, Hamburg, Germany) device was used to record a digital photoplethysmography pulse wave signal. A pulse oximetry sensor was placed on the index finger and the signal was recorded by a compact two-channel screening wrist worn device [7]. PSG and pulse oximetry signals were recorded in parallel for a total time in bed of 8 hours.

### Pulse wave analysis

PSG including sleep stages of 30-second epochs and respiratory events were evaluated and scored visually by certified sleep technicians according to 2012 AASM 2.0 criteria [29,30]. The overnight photoplethysmographic recording provided an unfiltered pulse wave signal during sleep. A specific quality signal was generated by the ChipOx pulse oximeter module (range 0–100%). A high quality refers to few motion artifacts and a high pulse wave amplitude due to strong pulsation. A signal quality of more than 85% was considered as artifact-free recording and used for the analysis [8]. In order to derive cardiovascular parameters and assess CV risk, an automated pulse wave analysis based on the Matching Pursuit algorithm was performed, a wavelet-related signal decomposition and feature extraction method [31]. This algorithm has been optimized and validated with a multicenter sleep cohort and was strongly associated with established CV risk factors based on the ESH/ESC CV risk matrix [4,8]. The signal was processed, denoised, and characteristic points of the signal were detected. Ten pulse wave parameters were extracted and computed: Pulse wave amplitude index, mean pulse propagation time (PPT), mean respiration-related pulse oscillation, pulse rate index, saturation of peripheral capillary oxygen index (SpO2-I), time of SpO2 below 90%, difference between pulse rate index and SpO2 index, periodic and symmetric desaturations, and irregular heart rate. For CV risk classification, these parameters were combined by a neuro-fuzzy system with nine rules. The overall CV risk (CRI, cardiac risk index) was scored on a scale from 0 (normal CV risk) to 1 (increased CV risk). The decomposition and classification process has been described in detail in previous studies [8].

For our analysis, we especially focused on overall CV risk and the single parameter vascular stiffness (PPT). The mean PPT of the complete recording time was reported. The PPT represents the time interval between the systolic peak and the subsequent reflected wave and provides a surrogate marker of pulse wave velocity and arterial stiffness. A shorter PPT indicates rigid and atherosclerotic vessels [32].

### Statistical analysis

Statistical analysis was performed using SPSS (IBM SPSS Statistics, Version 20) and R software language [33]. As most variables were not normally distributed, non-parametric tests (three-group Kruskal-Wallis test, two-group Mann-Whitney-U test, Spearman correlation) were applied and *p*-values <0.05 were considered statistically significant. To investigate differences between two groups, we performed the Mann-Whitney-U test. For differences between more than two groups, we first performed the Kruskal-Wallis test and then, individual Mann-Whitney-U tests to identify which groups differed significantly. Descriptive data were presented as median and interquartile range. In addition, a linear regression analysis with ordinary least squares for the main parameters including an interaction term of age and insomnia condition was used to investigate the influence of age. Due to the characteristics of a case-control study, participants were divided into two groups based on their insomnia condition: insomnia patients and non-insomnia participants. Insomnia subgroups were defined post-hoc based on objectively measured sleep efficiency (SE). By comparing the control group with both insomnia groups, a multiple comparisons alpha error may be possible. We present the uncorrected *p*-values in order to present independent trends between the insomnia groups. Result interpretation will be done based on the Bonferroni corrected alpha level of *p* = 0.025.

## Results

### Sample description

The final sample consisted of 26 healthy sleepers (controls: ISI Score < 8, Table 1) and 26 insomnia patients (IS group: 12 men (46%), median age = 35years (IQR 28–49), median BMI = 24kg/m^2^ (20–29), median ISI score = 18 (17–22)). Ten participants (5 insomniacs and 5 controls) displayed an AHI above 5 events/hour and three participants (2 insomniacs and 1 control) exhibited periodic leg movements with an index above 15 events/hour, displaying some signs of occult sleep apnea or motor dysfunction during sleep. Most insomnia patients presented moderately severe insomnia (65%, n = 17) and only few had severe insomnia (27%, n = 7) or mild insomnia (8%, n = 2). Thirty-one percent (n = 8) of the insomniacs had a history of more than six years. None of the insomnia patients had an established cardiovascular disease except two subjects in the control group, which had hypertension or a coronary heart disease. Baseline pulse rate and blood pressure were comparable. Insomniacs differed from controls regarding objective and subjective sleep quality with the insomnia patients presenting significantly poorer sleep (IS vs controls: median SE: 78% (IQR: 60–88) vs. 88% (83–93%), z(26/26) = -3.16, *p<0*.*01*; Total Sleep Time (TST): 376min (279–413) vs. 417min (398–441), z(26/26) = -3.102, *p<0*.*01*; Sleep Onset Latency to Stage 2 (SOL): 26min (21–41) vs. 19min (10–29), z(26/26) = -2.187, p = 0.03; Wake After Sleep Onset (WASO): 118min (55–125) vs. 57min (32–82), z(26/26) = -2,709, *p<0*.*01*; ISI score: 18 (17–22) vs. 1 (0–3), z(26,26) = -6.23, *p<0*.*001*; ESS score: 12 (8–15) vs. 5 (3–8), z(26,26) = -4.04, *p<0*.*001;* PSQI score:12 (10–14) vs. 3 (2–5), z(26,26) = -5.85, *p<0*.*001*).

**Table 1:** Sample description including polysomnography and pulse wave analysis parameters.

	IS + SE≤80%	IS + SE>80%	Controls	*p–*value^c^
	n = 14	n = 12	n = 26	
	**Anthropometric data**^b^			
**Men (n)**	8 (57%)	4 (33%)	14 (54%)	0.41
**Cardiovascular disease (n)**	0 (0%)	0 (0%)	2 (8%)	0.35
**Age (years)**	48.5 (38.0–53.8)	29.5 (24.3–33.8)	31.5 (28.0–47.3)	**0.02**
**BMI (kg/m**^**2**^**)**	23.2 (20.1–27.1)	23.9 (21.8–29.7)	22.4 (20.8–25.2)	0.59
**Alcohol (glasses/week)**	1.0 (0.8–1.0)	1.0 (0.0–1.0)	1.0 (0.8–2.0)	0.30
**Smoking (cigarettes/day)**	0.0 (0.0–0.0)	0.0 (0.0–0.0)	0.0 (0.0–0.0)	0.69
**Years since IS diagnosis**	5.5 (3.3–7.8)	5.0 (3.0–10.0)	n/a	0.81
***Persistent IS ≥ 6 years (n)***	5 (36%)	3 (25%)	n/a	
**Pulse rate (bpm)**^a^	70.0 (63.0–74.0)	75.0 (64.0–80.8)	64.0 (62.0–72.0)	0.13
**Systolic BP (mmHg)** ^a^	117.5 (100.0–130.0)	115.0 (100.3–128.8)	110.0 (105.0–126.3)	0.99
**Diastolic BP (mmHg)** ^a^	70.0 (60.0–80.5)	80.0 (64.0–83.8)	70.0 (70.0–80.0)	0.23
	**Subjective sleep quality (questionnaires)**^b^			
**ISI Score**	19.0 (17.8–22.3)	18.0 (15.3–19.0)	1.0 (0.0–3.3)	**0.00**
***No IS*, *ISI 0–7 (n)***	0 (0%)	0 (0%)	26 (100%)	
***Mild IS*, *ISI 8–14 (n)***	0 (0%)	2 (17%)	0 (0%)	
***Moderate IS*, *ISI 15–21 (n)***	8 (57%)	9 (75%)	0 (0%)	
***Severe IS*, *ISI 22–28 (n)***	6 (43%)	1 (8%)	0 (0%)	
**ESS Score**	10.0 (4.5–14.0)	13.0 (9.0–15.0)	5.0 (3.0–8.0)	**0.00**
***ESS > 10 (n)***	*7 (50%)*	*7 (58%)*	*0 (0%)*	
**PSQI Score**	13.0 (9.5–15.0)	12.0 (10.0–14.0)	3.0 (2.0–5.0)	**0.00**
***PSQI > 5 (n)***	*13 (93%)*	*12 (100%)*	*3 (12%)*	
**RLS-DI Score**	-14.0 (-16.0- -10.0)	-10.0 (-12.0- -1.5)	-19.0 (-19.0- -14.0)	**0.00**
	**Objective sleep quality (polysomnography)** ^b^			
**SE (%)**	67.1 (37.3–74.7)	88.1 (85.7–89.9)	88.0 (83.0–93.1)	**0.00**
**SOL (minutes)**	35.5 (11.7–55.0)	15.7 (5.9–19.7)	19.0 (9.9–28.9)	**0.02**
**TST (minutes)**	310.5 (180.3–361.1)	414.7 (409.6–424.1)	416.8 (397.5–440.9)	**0.00**
***TST ≥ 6 hours (n)***	4 (29%)	12 (100%)	23 (89%)	
**WASO (% of TST)**	16.1 (10.4–29.4)	14.4 (7.2–21.2)	16.5 (8.4–21.5)	0.38
**S1 (% of TST)**	18.8 (15.1–25.0)	15.3 (13.1–35.2)	19.0 (10.2–28.0)	0.80
**S2 (% of TST)**	44.6 (40.7–54.1)	48.5 (41.6–53.1)	45.1 (35.4–53.4)	0.67
**SWS (% of TST)**	20.3 (16.8–27.7)	23.0 (19.6–28.5)	22.4 (19.9–27.8)	0.47
**REM (% of TST)**	12.6 (8.6–16.2)	15.6 (13.1–35.2)	13.4 (8.9–14.9)	0.20
**Snoring (% of TST)**	18.0 (1.6–46.0)	13.9 (2.7–27.9)	15.6 (4.3–31.4)	0.92
**AHI (events/hour)**	1.5 (0.3–16.0)	1.3 (0.7–3.0)	1.3 (0.4–3.8)	0.84
***AHI 5–14 (n)***	*1 (7%)*	*1 (8%)*	*3 (12%)*	
***AHI ≥ 15 (n)***	*3 (21%)*	*0 (0%)*	*2 (8%)*	
**Oxygen desaturation index**	1.7 (0.5–22.7)	1.1 (0.4–2.5)	1.1 (0.3–2.6)	0.54
**Arousal index**	10.8 (6.7–15.6)	5.6 (5.1–10.7)	6.5 (4.7–8.4)	**0.04**
**PLMI (events/hour)**	11.9 (8.4–25.6)	2.1 (0.9–4.0)	2.1 (0.8–7.4)	
***PLMI > 15***	*2 (14%)*	*0 (0%)*	*1 (4%)*	
	**Cardiovascular parameters (pulse wave analysis)** ^**b**^			
**Pulse rate (average bpm)**	66.5 (57.5–70.0)	66.0 (63.0–68.5)	58.0 (51.8–63.3)	**0.01**
**PPT (ms)**	168.9 (147.4–232.0)	236.9 (214.5–254.0)	243.5 (179.6–284.2)	**0.01**
**Pulse RSA**	36.9 (30.6–44.9)	38.3 (34.1–49.3)	47.2 (33.6–60.6)	0.24
**PWA-I**	9.0 (6.8–10.5)	9.2 (8.5–10.8)	9.8 (7.5–11.1)	0.64
**CRI**	0.2 (0.0–0.7)	0.0 (0.0–0.4)	0.0 (0.0–0.3)	0.05

IS, insomnia; SE, sleep efficiency; BMI, body mass index; bpm, beats per minute; BP, blood pressure; mmHg, millimeters of mercury, unit for blood pressure; ISI, Insomnia Severity Index; ESS, Epworth Sleepiness Scale, scores above 10 indicate excessive daytime sleepiness and it is recommended to seek medical advice; PSQI, Pittsburgh Sleep Quality Index, scores above 5 indicate bad sleep quality; RLS-DI, Restless Leg Syndrome Diagnostic Index; SOL, sleep onset latency, stage 2; TST, total sleep time; WASO, wake time after sleep onset; S1, sleep stage 1; S2, sleep stage 2; SWS, slow-wave-sleep stage; REM, rapid-eye-movement sleep stage; AHI, apnea-hypopnea-index; PLMI, periodic leg movement index; PPT, pulse propagation time; ms, milliseconds; pulse RSA, pulse respiratory sinus arrhythmia; PWA-I, pulse wave amplitude index; CRI, cardiac risk index; n/a, not applicable.

^**a**^measured before polysomnography.

^b^Displayed are median (interquartile range) or number (proportion).

^c^Kruskal-Wallis test was used for continuous variables, Chi-square test for dichotomous variables. Significant values on 0.05 level are highlighted.

Due to the high range of objectively measured SE in the insomnia group (28.8% - 91.4% vs. control group: 66.4% - 97.1%), we decided to additionally divide the IS group into insomniacs with low SE≤80 (n = 14) and insomniacs with high SE>80% (n = 12) (Table 1). The three groups displayed a significant age effect (Kruskal Wallis Test: H(2) = 7.741, *p = 0*.*021*) with the low-SE insomniacs being significantly older than both, the insomniacs with high SE and the controls. While both IS groups still differed significantly from the control group regarding subjective sleep questionnaires, only the IS group with low SE differed significantly from the control group regarding objective sleep quality (Table 1). The low-SE insomniacs displayed significantly lower SE, shorter TST, and longer SOL than the other two groups. Only four of the low-SE insomniacs (29%) showed a TST of 6 hours or more. The high-SE insomniacs and the controls did not differ significantly with regard to these sleep parameters. As expected, there was also a higher arousal index in the low-SE group compared to the controls in the PSG recording (Table 1).

### Pulse wave analysis

**Fig 1:**
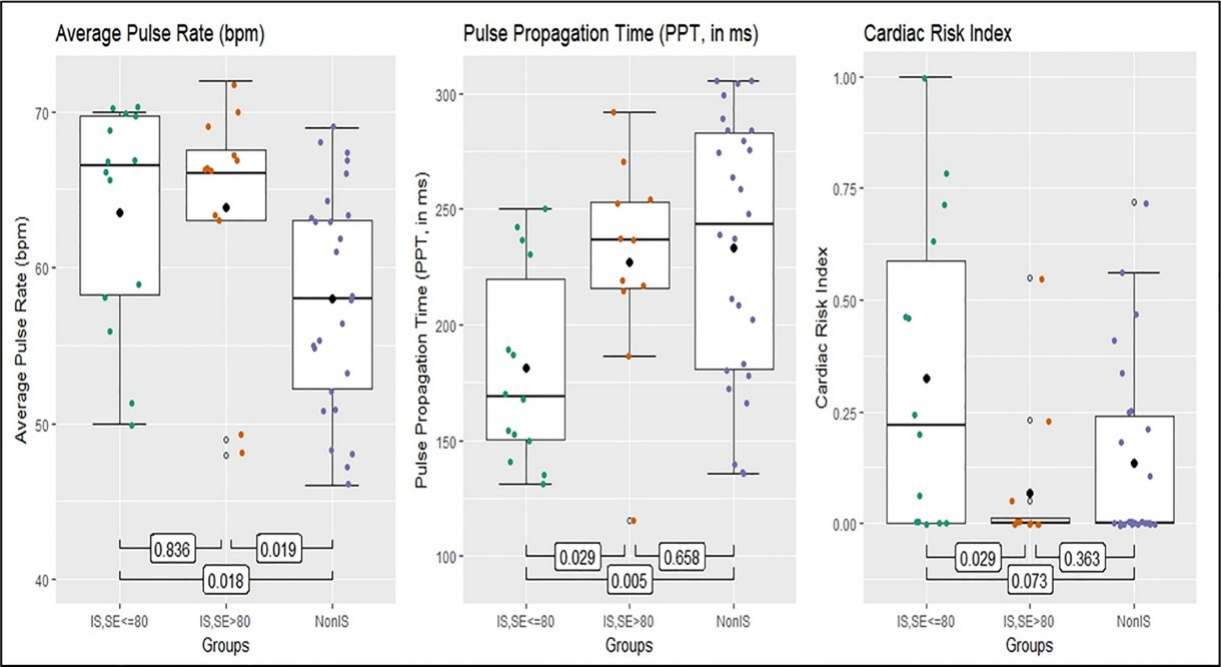
Pulse wave analysis parameters with group differences. IS, insomnia; SE, sleep efficiency. The boxes represent the interquartile range with the median, whiskers represent range of minimum and maximum value. Values outside are outliers of more than 3x interquartile range. *p*-values for group comparisons, performed with Mann-Whitney U test. Significant values on 0.05 level.

The pulse wave analysis revealed significant differences between insomnia and control groups with respect to average pulse rate (H(2) = 8.526, *p = 0*.*014*; Table 1 and Fig 1). The mean overnight pulse rate was higher for both IS groups compared with the control group (IS low-SE vs. controls: z(14,26) = -2.387, *p = 0*.*018*; IS high-SE vs. controls: z(12,26) = -2.364, *p = 0*.*019*) and did not differ significantly between the IS subgroups. This difference remained significant after control for age in a linear regression model (*p = 0*.*007*).

The single parameter PPT also differed significantly between groups (H(2) = 8.489, *p = 0*.*014*; Table 1 and Fig 1). Vascular stiffness–translating into reduced PPT–was elevated among low-SE insomniacs when compared with both controls and high-SE insomniacs (IS low-SE vs. IS high-SE: z(14,11) = -2.190, *p = 0*.*029*; IS low-SE vs. controls: z(14,26) = -2.751, *p = 0*.*005*, Fig 1). Insomnia with low SE tended to associate with increased vascular stiffness even after control for age as a strong predictor of PPT (linear regression, *p = 0*.*058*).

Further pulse wave derived parameters reflecting overnight hypoxia (desaturations, degree of profound hypoxia), skin sympathetic activity (pulse amplitude variability), or pulse rate variability did not differ between the insomnia groups and controls. More than 50% of the high-SE insomniacs and controls displayed a CRI of 0 indicating low overall CV risk in the composite cardiovascular risk evaluation. However, CRI was elevated in insomniacs with low SE compared to the other groups (H(2) = 5.842, *p = 0*.*054*, Table 1; and in IS low-SE vs. high-IS: z(14,11) = -2.211, *p = 0*.*029*; IS low-SE vs. controls: z(14,26) = -1.809, *p = 0*.*073*, Fig 1).

## Discussion

In our study we record nocturnal cardiovascular variables in insomnia patients in a novel manner. Signals derived from a pulse wave analysis during overnight PSG were compared between patients with insomnia and controls. The study provided three important findings. First, measures of cardiac and vascular sympathetic activity during sleep were increased in young and otherwise healthy insomnia patients compared with good sleepers. Second, we identified two subgroups of insomniacs, with and without objectively disturbed sleep, and observed that activation of vascular sympathetic tone was present only in individuals with an insomnia diagnosis based on subjective and objective criteria. Third, assessment of the pulse wave signal from pulse oximetry provided a feasible method to determine autonomic and cardiovascular parameters during sleep, even in patients with sleep disturbances.

### Pulse wave analysis during sleep

Our PSG recording and pulse wave analysis supported the notion of a physiological hyperarousal in insomniacs as previously demonstrated in terms of electroencephalographic features of disturbed sleep and elevated sympathetic tone [18,23,34]. Our findings are particularly relevant in light of a recent review that challenged the fundamental concept of an impaired heart rate variability and a physiological hyperarousal in insomnia due to lack of reproducibility [35]. The validated overnight pulse wave analysis detected signs of autonomic activity changes during nighttime and, as a surrogate marker of pulse wave velocity and arterial stiffness, the lower PPT in insomniacs with low SE suggested more rigid vessels during sleep. A composite variable, the Cardiac Risk Index, associated with conventional CV risk markers, was higher in insomniacs with low SE compared to insomniacs with objective good sleep and controls. These findings support a link between high autonomic tone, vascular stiffness and the appearance of insomnia at least in the low-SE group. Surrogate measures of autonomic activity may have yet unexplored applications in sleep medicine including insomnia classification and assessment of therapeutic interventions.

### Insomnia phenotypes and CV risk

We identified two insomnia phenotypes based on objective SE. While all insomniacs reported poor sleep, there were those with objectively good sleep (high-SE) and those with objectively disturbed sleep (low-SE). The insomniacs with low SE were associated with almost twice as long sleep latencies and wakefulness periods during sleep compared to good sleepers and high-SE insomniacs. Further, we identified differences in autonomic function in the insomnia subgroups suggesting that disturbances are particularly prominent in insomniacs with objectively verified poor sleep.

The association of insomnia and increased CV risk appears to depend on specific insomnia symptoms. There is still a lack of consensus regarding the nature of such symptoms [19]. Popular markers include long sleep latencies and low SE [23]. Others found CV differences in insomnia subgroups based on a combination of three symptoms: difficulties falling asleep, maintaining sleep, and waking up fatigued [12]. A sleep duration of less than 6 hours has been linked to higher risk of hypertension [15]. However, there are large differences regarding the individual need for sleep [16]. Parathasarathy et al. (2015) demonstrated that persistent (chronic) insomnia for at least 6 years was associated with increased CV risk [36]. Our low-SE insomniacs consisted of 70% with short sleep duration (less than 6 hours), half of the group presented severe insomnia, and one-third a persistent insomnia. None of our high-SE insomniacs had short sleep, only one presented a severe insomnia, and only three had a history of persistent insomnia. A cluster of specific insomnia symptoms including objective sleep quality, sleep time, insomnia severity, and duration of insomnia may result in even stronger differences in autonomic and cardiovascular function. Subsequent studies with insomnia phenotype clusters are recommended.

### Strengths and limitations

The cases and controls were carefully selected, and polysomnography was performed according to international standards; sleep data was evaluated by one scorer to exclude interscorer variability. The pulse wave technology was extensively validated in patients with and without sleep disorders [4,10]. The analysis used high-quality, artifact-free signal recordings to avoid movement artifacts [37]. The calculation of arterial stiffness during sleep by an oximeter-based pulse wave propagation time analysis was validated against applanation tonometry of the radial artery and assessment of aortic pulse wave velocity [4]. Pulse wave analysis has previously been applied for vascular function assessments in patients with sleep, cardiovascular, and respiratory disorders [32,38–40].

However, there are limitations. Participants with a previously known diagnosis of another sleep and sleep-related disorder including obstructive sleep apnea, restless leg syndrome and periodic leg movement disorder were excluded. Though, latent and unknown sleep apnea events and motor dysfunction during sleep are common findings in insomnia patients and in PSG in general according to the International Classification of Sleep Disorders by the American Association of Sleep Medicine [41]. Studies have shown that these symptoms may potentially cause autonomic activation, especially the combined presence of OSA and PLM [42,43]. However, we identified only few participants with an elevated AHI and PLMI and they were equally present in both groups, the insomniacs and controls, minimizing the potential influence. Also, we recorded only one night and could not account for habituation effects or spontaneous variability in sleep and autonomic function from night to night. In addition, our post-hoc analysis divided our small sample size of insomnia patients into two substantially smaller subgroups resulting in limited statistical power in the analysis. Also, age constituted a major confounder that may have influenced the interpretation of the results. Furthermore, as our insomnia patients were relatively young and healthy, potential cardiovascular changes caused by insomnia may not have been fully established. This may have led to an underestimation of the influence of insomnia on cardiovascular and autonomic function during sleep. Furthermore, pulse wave analysis while representing autonomic activity has its limitations. Differentiating between parasympathetic and sympathetic activity is difficult and may not completely reflect the outcome from skin or muscle sympathetic nerve recordings. Despite these limitations, our results–while not a definite confirmation–propose a clear scientific value as they generate a hypothesis, which needs validation in larger and age-matched cohorts. Future studies are also needed to elucidate if medication or therapy in insomnia may affect markers of CV risk in insomniacs with low SE.

### Clinical application

Our findings emphasized the negative effect of insomnia on autonomic function already in young and cardiovascular healthy people, and therefore, its link to elevated long-term CV risk. In particular, these changes affected insomnia patients with subjectively defined and objectively assessed poor sleep. This has important implications for insomnia phenotypes, including paradoxical insomnia defined as subjective insomnia without objective manifestation. In order to effectively differentiate and treat these insomnia phenotypes, a functional assessment of autonomic biomarkers may be useful. Improved phenotypic classification, including overnight characterization of autonomic and CV function, may lead to an altered clinical practice better tailored to address individualized therapy.

### Conclusion

Our study indicated to use non-invasive overnight pulse wave analysis for assessment of relevant autonomic and cardiovascular function during sleep in insomniacs. Findings suggested a hyperarousal state in insomnia patients that may reflect an elevation of cardiovascular risk.

## Supporting information

S1 File Masterspreadsheet with raw data information. (PDF)
