# Supplementary material for: Overnight pulse wave analysis to assess autonomic changes during sleep in insomnia patients and healthy sleepers
Source: PLoS One. 2020 May 7;15(5):e0232589. doi: 10.1371/journal.pone.0232589 (PMC7205215; doi:10.1371/journal.pone.0232589)
Supplement: S1 File — (PDF) [file pone.0232589.s001.pdf]

Participant	Age	ISI Score	Meds	Sex	BMI	SBP	DBP	PR_pre	alcohol	smoking	ESS	RLS-DI	PSQI	TIB	TST	SleepEfficiency	SleepLatency_1	SleepLatency_2	SleepLatency_3	AHI	RDI_PSG	WASO	REM	SleepStage1
IM_01 I	36	16	L-Thyroxin	2	24,8	110	90	64	1	0	6	-10	12	07:37:20	06:57:29	91,3	00:05:40	00:08:10	00:25:10	1,1	1,7	00:39:50	01:16:30	01:17:00
IM_02 I	53	18		1	26,2	115	70	76	2	0	8	-14	13	08:01:10	06:11:30	77,2	00:03:35	00:06:05	00:12:35	24,4	25,2	01:49:40	00:52:00	00:44:00
IM_03 G	29	0		1	25,9	130	80	60	1	0	8	-17	2	07:58:45	07:06:15	89,6	00:00:00	00:09:20	00:28:50	3,1	3,4	00:49:37	01:36:25	01:19:50
IM_05 I	48	22		1	20,1	88	55	80	0	0	13	-12	15	08:21:49	02:24:30	28,8	00:19:20	00:33:20	06:27:20	1,3	1,3	05:57:19	00:10:00	00:48:30
IM_06 I	60	15		2	22,3	110	60	70	1	0	2	-19	3	08:05:55	06:00:59	74,6	00:35:56	00:45:56	01:06:56	0,3	0,5	02:03:06	01:29:30	00:56:29
IM_07 G	30	0	L-Thyroxin 50	2	22,4	110	70	62	2	0	3	-19	5	07:54:50	05:15:30	66,4	00:38:20	00:52:50	02:45:50	0,0	0,8	02:39:20	00:29:00	01:17:30
IM_08 G	36	0		2	22,1	120	70	64	1	0	9	-15	2	07:38:39	06:37:58	86,8	00:08:09	00:16:39	00:23:39	0,3	0,3	01:00:39	00:49:00	01:13:00
IM_09 G	44	5	-	1	21,2	105	75	74	1	0	3	-19	3	07:20:48	05:17:30	72	00:31:03	00:39:03	01:19:33	1,9	1,9	02:03:18	00:30:30	01:00:00
IM_10 G	45	0	Ramipril hexal Amlodipin	1	36,6	140	80	82	2	0	6	-19	3	07:57:15	06:43:45	84,6	00:06:00	00:09:30	00:39:30	24,0	24,0	01:13:30	00:27:30	01:45:15
IM_15 I	45	24	-	2	16,8	120	70	52	1	0	11	-12	17	08:00:00	03:03:00	38,1	01:23:00	01:40:00	04:11:30	0,0	0,0	04:56:59	00:21:30	01:34:00
IM_19 I	28	15	-	1	21	130	80	64	1	0	8	-9	10	07:53:30	07:01:30	89,7	00:17:59	00:21:59	00:38:29	2,8	3,0	00:48:16	00:54:00	00:41:30
IM_20 I	33	18	-	1	35,8	120	80	80	0	0	15	-16	10	08:03:20	06:42:00	83,2	00:13:30	00:21:00	00:50:00	0,7	0,7	01:21:20	00:22:30	01:38:00
IM_21 I	57	21	-	2	22,8	150	80	72	0	0	15	-12	12	07:56:59	06:49:59	86	00:19:29	00:20:59	00:28:59	0,6	0,7	01:06:59	01:02:29	01:21:29
IM_22 G	29	4	-	1	25,9	100	50	64	0	0	10	-17	8	08:00:30	07:44:30	96,7	00:03:59	00:06:59	00:17:29	5,2	5,2	00:15:59	01:12:00	01:02:00
IM_24 G	25	0	-	2	22,3	105	70	72	0	0	6	-19	2	08:19:49	06:41:00	80,2	00:22:29	00:27:29	00:38:29	0,7	0,9	01:38:49	00:52:00	00:55:00
IM_26 I	32	23	-	1	20,1	100	60	67	1	0	14	3	13	07:58:59	03:31:00	44,1	02:37:30	03:14:00	04:30:00	1,2	1,2	04:27:59	00:04:00	01:32:00
IM_28 G	31	0	L-Thyroxin 37,5	2	20,2	130	75	72	1	0	3	-13	2	07:46:03	07:18:32	94,1	00:00:00	00:03:07	00:12:07	0,7	1,8	00:27:30	01:25:59	00:59:37
IM_29 G	42	3	-	2	24	105	70	62	0	0	3	-5	5	08:28:16	06:36:00	78,2	00:17:46	00:33:16	00:52:46			01:50:14	01:05:00	00:56:30
IM_30 G	48	2	Pille	2	29,1	120	80	67	2	0	4	-19	6	08:01:39	07:02:00	87,6	00:04:59	00:23:29	00:41:30	13,3	13,3	00:59:39	01:02:30	03:22:00
IM_31 G	30	0	-	1	25,1	110	70	60	2	0	5	-19	2	07:48:10	06:51:30	87,9	00:03:30	00:11:30	00:31:00	3,2	3,2	00:56:40	00:55:30	01:47:00
IM_32 G	54	3	-	1	24	130	80	62	1	0	1	-19	4	07:57:39	06:28:00	81,2	00:33:49	00:37:19	00:59:19	1,1	1,1	01:29:39	01:31:30	00:44:00
IM_33 I	40	22	L-Thyroxin 50	2	20,2	100	65	72	1	0	9	-16	14	08:08:35	06:01:30	74,3	00:14:00	00:23:30	00:45:00	1,6	4,5	02:04:50	00:37:00	01:26:00
IM_34 G	56	1	-	1	29	125	80		1	0	8	-19	4	07:59:59	06:41:30	83,6	00:13:29	00:22:59	00:31:59	21,6	21,6	01:18:29	00:47:00	02:33:30
IM_35 G	48	4	-	2	19,7	110	80	62	1	0	9	4	6	08:03:59	07:49:59	97,1	00:05:29	00:09:29	00:31:59	0,1	0,1	00:13:59	01:20:00	00:51:30
IM_36 G	32	0	-	2	22,3	80	50	72	1	0	8	n.a.	5	07:59:43	07:26:27	93,1	00:10:59	00:17:59	00:31:59	0,6	1,7	00:33:13	01:46:30	00:57:30
IM_40 G	55	4	-	2	23,8	120	80	74	0	0	5	-19	3	08:00:59	07:19:00	91,9	00:05:59	00:09:59	00:21:29	10,5	11,1	00:38:42	01:24:30	01:11:30
IM_41 I	56	16	-	1	35,5	140	85	72	1	0	8	-14	10	07:48:00	04:39:00	60,1	00:35:30	00:39:00	00:58:30	30,8	31,2	01:47:07	00:41:30	02:19:30
IM_42 I	49	18	-	2	24,1	140	85	68	1	0	5	-14	8	07:45:59	05:45:00	74,5	00:49:00	01:04:00	01:19:30	2,3	3,1	01:58:21	00:55:00	01:44:30
IM_45 G	32	0	-	1	25,3	120	70	68	2	0	5	-15	1	08:05:00	07:07:00	88	00:24:30	00:45:00	00:58:00	0,3	0,3	00:58:00	01:06:30	01:26:00
IM_46 G	31	5	-	1	24,5	110	70	60	0	0	5	-14	2	08:05:59	06:32:29	80,8	00:37:29	00:56:29	01:01:59	1,5	1,7	01:33:26	00:27:00	01:43:00
IM_47 G	18	1	-	2	15,1	100	55	62	0	0	1	-14	3	08:08:59	06:49:00	83,6	00:05:59	00:23:29	00:37:29	0,0	0,0	01:19:59	00:56:00	01:23:30
IM_48 G	19	1	-	1	20,3	130	70	70	1	0	1	-19	3	08:07:00	06:49:30	84,8	00:14:30	00:25:30	00:30:00	1,3	1,3	01:13:20	00:55:00	01:07:00
IM_49 G	19	3	-	2	19,8	105	60	72	1	0	9	-19	3	08:06:59	07:39:30	94,4	00:08:29	00:19:29	00:31:59	1,1	1,4	00:27:29	00:47:00	00:57:00
IM_50 G	19	2	Pille	2	17,3	110	70		1		3	-14	3	04:56:28	04:35:58	93,1	00:13:00	00:18:30	00:25:00	0,5	2,1	00:20:30	00:14:58	00:24:30
IM_51 G	19	0	-	1	22,2	110	75	60	1	0	1	-19	4	07:51:39	07:07:00	90,5	00:10:29	00:18:29	00:25:59	2,1	2,1	00:44:39	00:34:30	01:37:00
IM_52 I	21	18	-	1	29	130	80	80	0	7,5	14	-14	11	08:01:29	05:54:30	73,9	00:35:29	01:24:59	01:29:29	13,2	13,2	02:05:02	00:51:30	01:37:00
IM_53 I	22	18	-	2	21,8	100	70		1	0	10	3	12	08:00:00	07:18:30	91,4	00:12:00	00:24:30	00:31:30	1,0	1,1	00:41:30	01:00:30	00:37:30
IM_54 I	21	19	Pille	2	18,7	105	65	62	1	0	13	-10	15	07:42:49	05:42:00	75	00:04:19	00:16:49	00:33:19	1,2	1,6	01:54:10	00:45:00	01:47:30
IM_55 I	28	18	Pille	2	29,9	120	85	80	0	0	15	-10	14	08:03:15	06:58:00	87	00:19:45	00:21:15	00:32:15	2,9	5,0	01:02:20	00:56:00	00:44:30
IM_56 I	49	17	-	2	26,4	120	80		0	0	17	-16	5	08:07:37	06:25:00	79	01:13:00	01:14:30	01:25:00	0,0	0,3	01:42:32	00:21:00	01:39:30
IM_57 I	31	17	-	2	19,2	101	62	65	2	0	13	-19	8	08:04:59	07:06:00	88,2	00:04:59	00:09:29	00:17:29	1,4	2,8	00:56:51	01:03:30	00:46:30
IM_59 G	47	0	-	1		130	80	62	1	0	5	-21	3	08:03:29	07:41:30	95,4	00:04:29	00:14:29	00:28:29	0,1	0,4	00:21:59	01:10:30	00:40:30
IM_61 G	31	0	-	1	21,9	110	65	62	2	1	8	-1	2	07:50:09	07:14:30	92,4	00:09:30	00:20:30	00:25:00	2,9	3,0	00:35:39	01:34:00	01:01:00
IM_62 I	50	22	-	1	30,6	130	82	58	1	0	3	-21	17	08:00:39	04:37:00	58	00:24:21	00:27:51	01:15:21	0,2	0,2	03:20:28	00:10:00	00:49:30
IM_63 I	28	19	Pille	2	21,8	100	60		1	0	13	1	15	08:03:00	07:05:00	88,7	00:20:00	00:35:00	00:42:00	0,7	0,8	00:53:41	00:43:00	00:37:30
IM_65 G	56	5	ASS 100 Efient 10 Simvastatin 20	1	23,2	120	70	76	2	0	8	-15	5	08:07:59	07:28:30	92,6	00:07:29	00:08:29	00:13:59	4,3	4,4	00:35:56	01:01:00	00:32:30
IM_67 I	34	13	-	1	29	125	85	83	0		16	-10	12	07:48:12	06:51:00	88	00:06:31	00:13:31	00:21:31	3,3	3,6	00:55:40	00:50:30	01:22:00
IM_68 I	22	19	-	2	23,4	100	60	78	1		16	-11	14	07:37:46	06:52:00	90	00:03:30	00:39:30	00:49:00	0,1	0,1	00:45:42	00:55:30	00:29:00
IM_69 I	47	24	-	1	20,1	88	58	64	2	0	0	-10	11	07:59:59	02:37:29	34	00:35:30	00:36:30	03:05:00	4,2	4,2	05:06:11	00:14:30	00:58:29
IM_71 I	23	24	Mexin	2	24,4	110	80	58	0		9	1	15	08:00:39	06:49:30	85,2	00:18:59	00:23:59	00:34:59	3,0	3,0	01:11:09	00:41:30	01:00:00
IM_73 I	31	11	-	1	32,5	140	80	106	1		9	-12	9	07:30:20	06:20:00	85,6	00:22:50	00:35:20	00:43:50	9,5	11,8	01:03:51	01:15:30	01:52:30
IM_75 I	62	19	-	1	24,6	125	70	70	1		15	3	14	08:15:04	02:52:00	34,7	00:04:44	00:22:14	00:25:14	36,6	36,6	00:20:14		01:12:00

SleepStage2	SleepStage1-2	SleepStage3-4	WASO_%	REM_%	SleepStage1_%	SleepStage2%	SleepStage1-2_%	SleepStages3-4_%	ODI_PSG	Sp02_min_PSG	Sp02_basal_PSG	TimeBelow90_PSG	HR_max	HR_min	HR_average	Arousal_n	Arousal_Index	Resp_Arousal	HR_Arousal	Spont_Arousal	snoring	Recording_PulseGood
03:02:30	04:19:30	01:21:29	8,7	18,3	18,4	43,7	62,2	19,5	0,3	94	97		116	51	64	56	8,1	0	64,3	21,4	17,9	07:40:15
03:49:30	04:33:30	00:46:00	22,8	14,0	11,8	61,8	73,6	12,4	18,3	85	91	00:41:03	72	41	49	81	13,1	13,6	0	28,4	52,2	07:44:02
02:29:30	03:49:20	01:40:30	10,4	22,6	18,7	35,1	53,8	23,6	1,7	92	95		90	47	62	56	7,9	5,4	57,1	23,2	3,8	07:28:38
01:01:30	01:50:00	00:24:30	71,2	6,9	33,6	42,6	76,1	17,0		92	95		83	54	64	14	5,8	7,1	21,4	57,1	5,6	07:19:32
02:44:00	03:40:29	00:51:00	25,3	24,8	15,7	45,4	61,1	14,1	0,3	94	97		93	57	69	61	10,2	1,6	8,2	45,9	25,5	07:43:41
02:15:30	03:33:00	01:13:30	33,6	9,2	24,6	42,9	67,5	23,3		86	96	00:00:30	90	49	62	16	3	0	25	56,3	17,1	07:41:51
03:22:59	04:35:59	01:12:59	13,2	12,3	18,3	51,0	69,3	18,3		95	97		78	44	51	37	5,6	0	0	62,2	81,5	07:42:31
02:52:00	03:52:00	00:55:00	28,0	9,6	18,9	54,2	73,1	17,3	0,6	91	96		95	54	66	37	7	0	5,4	40,5	6,9	07:33:54
03:38:00	05:23:15	00:53:00	15,4	6,8	26,1	54,0	80,1	13,1	18,3	85	96	00:01:15	95	53	66	66	9,8	13,6	12,1	31,8	30,5	07:42:01
00:52:00	02:26:00	00:15:30	61,9	11,7	51,4	28,4	79,8	8,5		82	96	00:14:31	74	44	52	5	1,8	0	40	60	0	07:08:16
03:58:00	04:39:30	01:28:00	10,2	12,8	9,8	56,5	66,3	20,9	1,4	93	96		77	39	48	39	5,6	5,1	71,8	15,4	48,8	07:47:15
01:57:00	03:35:00	02:44:30	16,8	5,6	24,4	29,1	53,5	40,9		93	96		89	53	66	24	3,6	0	4,2	70,8	43	07:38:29
02:57:00	04:18:29	01:29:00	14,0	15,2	19,9	43,2	63,0	21,7	0,4	90	96		88	38	49	36	5,6	0	5,6	83,3	1,7	07:44:48
03:55:30	04:57:30	01:35:00	3,3	15,5	13,3	50,7	64,0	20,5		97	99		88	40	51	52	6,7	0	51,9	40,4	8,5	07:39:45
03:34:30	04:29:30	01:19:30	19,8	13,0	13,7	53,5	67,2	19,8		94	97		105	51	66	46	6,9	0	37	45,7	26,4	08:12:52
01:43:30	03:15:30	00:11:30	55,9	1,9	43,6	49,1	92,7	5,5	0,6	93	96		88	48	64	54	15,5	1,9	38,9	53,7	6,3	07:15:45
03:30:00	04:29:37	01:22:56	5,9	19,6	13,6	47,9	61,5	18,9		95	97		89	45	52	34	4,7	0	2,9	94,1	4,9	07:44:47
03:26:00	04:22:30	01:08:30	21,7	16,4	14,3	52,0	66,3	17,3		84	94	02:01:50	107	45	57	4	0,6	0	25	50	0,1	06:43:47
01:26:00	04:48:00	01:11:30	12,4	14,8	47,9	20,4	68,2	16,9	2,3	86	94	00:20:22	94	58	67	34	6,7	14,7	14,7	44,1	27,1	07:43:41
02:48:30	04:35:30	01:20:30	12,1	13,5	26,0	40,9	67,0	19,6	1,5	92	96		86	43	54	68	10,1	0	36,8	41,2	26,4	04:36:02
02:51:30	03:35:30	01:21:00	18,8	23,6	11,3	44,2	55,5	20,9	0,2	93	97		90	45	62	36	5,6	5,6	2,8	75	35,3	06:25:33
03:27:30	04:53:30	00:31:00	25,6	10,2	23,8	57,4	81,2	8,6	0,2	92	97		107	62	70	305	52,7	0,7	3	94,1	0,2	05:34:04
02:31:30	05:05:00	00:49:30	16,4	11,7	38,2	37,7	76,0	12,3	18,2	85	96	00:03:30	81	54	62	120	17,9	25,8	0	30	82,4	07:41:47
04:12:59	05:04:29	01:25:30	2,9	17,0	11,0	53,8	64,8	18,2		93	96		94	52	65	57	7,3	0	22,8	66,7	0,2	05:53:06
03:36:27	04:33:57	00:56:00	6,9	23,9	12,9	50,7	63,6	12,5	0,3	93	97		94	45	57	43	5,9	0	14	86	1,6	06:42:59
03:12:00	04:23:30	01:31:00	8,0	19,2	16,3	43,7	60,0	20,7	11,1	83	92	00:55:33	81	39	47	89	12,2	19,1	3,4	38,2	22,2	07:20:07
01:11:30	03:31:00	00:26:30	22,9	14,9	50,0	25,6	75,6	9,5	27,1	84	97	00:04:01	121	60	69	104	22,4	46,2	1	13,5	51,7	05:44:09
01:57:00	03:41:30	01:08:30	25,4	15,9	30,3	33,9	64,2	19,9	1,7	91	96		93	52	66	91	15,8	0	4,4	56	24,8	07:40:58
03:12:30	04:38:30	01:22:00	12,0	15,6	20,1	45,1	65,2	19,2	0,1	91	95		90	48	57	58	8,2	1,7	34,5	46,6	46,9	08:01:35
03:07:59	04:50:59	01:14:29	19,2	6,9	26,2	47,9	74,1	19,0	0,9	92	96		89	43	52	57	8,8	0	31,6	57,9	0,1	07:43:03
03:04:00	04:27:30	01:25:30	16,4	13,7	20,4	45,0	65,4	20,9	2,8	80	89	04:28:24	92	42	54	21	3,1	0	57,1	33,3	0	06:44:33
02:18:00	03:25:00	02:29:30	15,1	13,4	16,4	33,7	50,1	36,5	0,2	81	97	00:01:08	87	37	46	39	5,7	0	66,7	28,2	7,6	07:40:10
03:01:30	03:58:30	02:54:00	5,6	10,2	12,4	39,5	51,9	37,9	0,7	83	94	00:00:08	95	46	63	48	6,3	0	35,4	45,8	19,6	07:44:55
02:28:30	02:53:00	01:28:00	6,9	5,4	8,9	53,8	62,7	31,9		93	99		1	51	70	21	4,6	0	52,4	28,6	4,5	07:42:43
03:03:00	04:40:00	01:52:30	9,5	8,1	22,7	42,9	65,6	26,3	0,3	96	99		86	44	55	89	12,5	6,7	59,6	28,1	5,3	06:43:02
01:55:30	03:32:30	01:30:30	26,0	14,5	27,4	32,6	59,9	25,5	8,5	86	97	00:02:53	109	40	52	76	12,9	23,7	28,9	19,7	1,8	07:41:21
03:07:00	03:44:30	02:33:30	8,6	13,8	8,6	42,6	51,2	35,0	0,4	95	98		91	61	67	40	5,5	0	2,5	80	0,3	06:10:16
02:05:00	03:52:30	01:04:30	24,7	13,2	31,4	36,5	68,0	18,9	0,7	82	93	01:43:25	124	56	68	57	10,1	0	33,3	29,8	68,4	04:37:54
03:23:00	04:07:30	01:54:30	12,9	13,4	10,6	48,6	59,2	27,4	2,3	90	97		97	53	68	76	10,9	7,9	21,1	52,6	5,8	06:07:53
03:02:30	04:42:00	01:22:00	21,0	5,5	25,8	47,4	73,2	21,3		93	96		80	60	67	45	7	0	0	73,3	44,1	07:44:01
03:47:00	04:33:30	01:29:00	11,7	14,9	10,9	53,3	64,2	20,9	0,7	92	97		107	50	66	35	4,9	2,9	14,3	71,4	8,6	07:44:26
04:21:00	05:01:30	01:29:30	4,6	15,3	8,8	56,6	65,3	19,4		93	96		82	39	48	16	2,1	0	12,5	62,5	39,6	07:21:34
02:52:00	03:53:00	01:47:30	7,6	21,6	14,0	39,6	53,6	24,7	1,1	92	94		124	32	49	24	3,3	4,2	41,7	50	14,1	07:44:36
02:42:00	03:31:30	00:55:30	41,7	3,6	17,9	58,5	76,4	20,0		94	96		97	53	65	46	10,0		43,5	50,0	11,2	08:23:21
03:47:30	04:25:00	01:57:00	11,1	10,1	8,8	53,5	62,4	27,5		94	96		99	53	63	34	4,9	2,9	14,7	58,8	21,4	07:44:08
03:27:30	04:00:00	02:27:30	7,4	13,6	7,2	46,3	53,5	32,9	2,3	93	96		87	54	64	42	5,6	9,5	16,7	23,8	34,1	08:02:13
03:40:30	05:02:30	00:58:00	11,9	12,3	20,0	53,6	73,6	14,1	2,9	85	95	00:42:58	86	41	63	84	48,1	2,4	10,7	73,8	30,1	04:24:14
03:29:00	03:58:00	01:58:30	10,0	13,5	7,0	50,7	57,8	28,8	0,1	94	97		97	45	66	38	5,5		57,9	39,5	0,4	05:50:43
00:56:30	01:54:59	00:28:00	63,8	9,2	37,1	35,9	73,0	17,8		95	97		69	41	48	7	2,7		42,9	57,1	0,8	07:47:20
03:46:00	04:46:00	01:22:00	14,8	10,1	14,7	55,2	69,8	20,0	1,6	92	97		107	55	73	80	11,9		18,8	77,5	10,7	07:57:12
01:47:00	03:39:30	01:25:00	14,2	19,9	29,6	28,2	57,8	22,4	7,3	86	95	01:29:35	103	48	70	63	10,0	6,3	17,5	36,5	17,1	07:13:15
00:56:00	02:08:00	00:44:00	4,1	0,0	41,9	32,6	74,4	25,6	32,4	79	94	00:05:37	77	48	60	32	11,3	43,8	3,1	9,4	38,4	08:45:00

CRI	RDI_PO	ODI_PO	Sp02_min_PO	Sp02_avg_PO	TimeBelow90	PR_PO	AAI	irregularePulse	RCRD	low_PRV	low_PWV	short_PW_RT	PASD	FDS	LBS	PPT_INDEX	PULS_INDEX	PULS_RSA	PWA_INDEX_10_20	PWA_INDEX_20_30	PWA_INDEX_30_40	PWA_INDEX_40_50	PWA_INDEX_50_60	PWA_INDEX_60_70	PWA_INDEX_70_80	PWA_INDEX_80_100
0,55	1	1,6	88	94	00:00:16	69	19,7	0	0	0,3	0,7	1	0	0	0											
0,46	5,6	3	86	93	00:15:04	50	20,2	0	0	0,3	0	0,5	0,3	0	0	152,68	24,61	36,62	22,54	24,61	19,69	12,17	3,1	0,12	0	0,12
0	0,9	0,4	93	97	00:00:00	61	29,2	0	0	0	0	0	0	0	0	289,44	47,6	53,2	33,79	28,96	17,03	11,93	5,76	2,27	0,26	0
0	1,5	1	77	96	00:00:16	70	18,7	0	0	0	0,5	0	0	0	0	230,41	31,25	37,19	11,8	13,33	9,58	7,36	5	0,69	0,55	0
0,63	0,8	0,5	89	97	00:00:04	70	60,9	0	0	0	0,7	1	0	0	0	131,18	41,44	32,74	10	7,79	11,56	13,25	22,86	21,95	4,93	0
0	0,5	0,4	90	98	00:00:00	63	29,9	0	0	0,2	0,7	0	0	0	0	263,61	25,2	33,88	13,45	8,74	6,79	8,48	10,57	12,53	3,39	0,13
0	0	0	88	97	00:00:07	51	28,9	0	0	0,4	0,7	0	0	0	0	247,83	21,97	56,29	16,25	7,8	6,89	10,4	9,23	11,7	4,03	0
0,21	0,7	0,5	87	98	00:00:03	67	11,8	0	0	0,6	0,8	0	0	0	0	177,79	14,99	25,02	16,18	9,28	5,57	5,17	2,38	0,66	0,39	0,92
0,18	4	2,1	89	97	00:00:12	66	40,5	0	0	0	0,5	0	0	0	0	180,2	59,42	34,9	12,87	10,53	14,82	16,64	12,48	7,15	2,34	0
0,46	1,8	2	83	97	00:00:49	56	17,2	0	0	0,2	0,7	0	0	0	0	167,79	27,15	39,12	10,83	9,56	7,45	4,22	4,5	2,39	1,12	1,26
0	0,9	0,5	91	97	00:00:00	48	32,5	0	0	0,3	0,8	0	0	0	0	253,97	27,82	79,84	12,88	6,69	5,66	6,56	9,66	8,37	7,34	1,54
0	5,1	3,3	77	96	00:00:37	66	25,5	0	0	0,2	0,3	0	0	0	0	292,18	29,7	26,12	20,89	12,35	16,56	13,27	8,28	3,15	0,26	0,39
0,23	1,7	0,9	93	97	00:00:00	49	35,9	0	0	0	0,4	1	0	0	0	115,59	39,65	43,39	9,29	12,78	11,62	10,59	9,17	2,58	0,51	0,12
0	0	0	95	97	00:00:00	51	48,7	0	0	0	0,4	0	0	0	0	305,68	64,11	63,9	19,45	11,75	11,62	13,18	15,14	11,09	4,04	0,26
0,11	0,5	0,2	92	97	00:00:00	68	44,2	0	0	0	0,8	0	0	0	0	183,03	48,47	48,19	10,23	8,03	10,23	12,17	14,37	12,05	4,99	0,36
0,06	1,2	0,8	87	96	00:00:06	67	42,8	0	0	0	0,3	0,6	0	0	0	149,59	76,94	53,28	10,22	12,98	10,08	12,15	12,29	7,18	1,93	0,27
0,41	0,8	0,8	81	98	00:00:18	52	45,8	0	0	0,4	0,9	0,8	0	0	0	136,6	26,72	60,26	5,8	6,97	9,29	13,04	17,94	15,49	14,58	2,58
0,47	0,3	0,1	83	97	00:00:11	56	25,7	0	0	0	0,7	0,3	0	0	0	166,21	35,16	46,19	11,02	4,91	5,51	5,51	8,04	7,3	3,72	0,14
0,72	3,6	2,5	87	96	00:00:22	67	48	0	0	0	0,8	0,9	0	0	0	135,67	37,2	26,99	7,12	10,5	7,77	11,01	19,44	16,33	7,12	1,16
0	1,7	0,9	89	96	00:00:02	55	55,6	0	0	0	1	0	0	0	0	299,48	61,88	61,72	7,51	5,52	3,09	8,17	11,49	22,98	12,59	1,76
0,34	1,2	1,4	85	96	00:00:49	62	17,3	0	0	0,8	0,7	0,1	0	0	0	172,73	8,75	24,54	13,9	8,59	7,65	10,15	9,68	6,71	2,65	0
1	0	0	90	97	00:00:00	70										187,21	12,07	18,23	27,56	16,57	9,36	9,54	9,72	5,58	4,86	0,18
0,25	7,1	6,2	86	95	00:01:42	63	39,4	0	0	0	0,6	0	0,1	0	0	202,57	39,07	32,86	9,5	14,97	16,54	17,32	12,63	6,38	1,95	0,91
0	2	2,2	72	97	00:05:54	64	8,3	0	0	0,7	0,4	0	0	0	0	284,16	11,85	28,94	15,29	10,99	6,35	2,92	1,54	0,85	1,03	0,34
0	0,7	0,4	91	97	00:00:00	58	57,2	0	0	0,2	0,5	0	0	0	0	284,22	23,99	43,87	11,47	11,02	14	19,96	19,07	20,11	8,19	0,74
0,25	7,9	7	84	94	00:02:45	47	25,4	0	0	0	0,5	0	0,1	0	0	211,52	36,71	45,03	15,31	12,28	8	11,59	8,28	3,45	0,41	0,41
0,2	22,4	20,6	78	96	00:10:22	69	38,9	0	0	0	1	0	0,1	0	0	189,09	41,58	32,92	6,14	4,21	5,26	12,1	12,1	11,23	5,79	2,28
0,78	2	1,3	82	96	00:00:09	66	32	0	0	0,2	1	0,8	0	0	0	140,83	33,83	31,14	8,09	3,65	7,18	9,4	12,93	11,1	3,13	0,52
0	0,4	0	92	95	00:00:00	58	31,9	0	0	0	0	0	0	0	0	279,52	49,86	49,92	27,11	13,12	12,12	15,37	13,24	4,49	0,49	0
0	7,6	5,8	78	97	00:01:58	53	42,1	0	0	0	0,3	0	0	0	0	239,17	71	57,21	14,69	11,31	15,34	12,48	8,32	5,46	2,86	1,43
0	11	9,3	82	96	00:09:56	55	23,6	0	0	0,1	0,7	0	0	0	0	305,52	35,22	73,75	11,29	7,28	3,71	4,75	6,09	7,58	1,93	1,93
0	0,3	0	88	97	00:00:04	46	27,4	0	0	0,4	0,1	0	0	0	0	274,35	24,04	70,21	21,17	14,11	13,33	16,07	11,24	3	0,91	0,13
0	0,4	0	94	96	00:00:00	63	54,1	0	0	0	0,2	0	0	0	0	275,57	55,65	51,06	13,42	17,3	14,97	15,62	16,52	13,81	5,42	0,12
0	1,4	1,6	78	98	00:02:10	69	47,3	0	0	0,6	0,6	0	0	0	0	208,79	13,54	27,67	11,45	10,67	7,94	8,59	12,37	23,69	19,01	3,77
0	5,1	3,4	84	97	00:00:34	55	51,1	0	0	0	0,5	0	0	0	0	237,47	44,59	69,17	10,44	12,23	16,1	18,04	18,79	11,78	6,71	2,23
0	5,5	3,5	88	96	00:00:30	58	39,4	0	0	0	0,3	0	0	0	0	236,76	32,06	73,04	12,82	12,56	11,51	11,51	9,94	10,2	7,98	0,39
0	2,8	2,3	83	97	00:00:37	67	32,2	0	0	0,8	0,4	0	0	0	0	252,59	5,84	27,23	12,5	9,42	9,09	8,93	13,15	13,48	7,3	2,11
0	0,9	1,1	64	97	00:00:28	70	30,4	0	0	0	1	0	0	0	0	250,48	33,84	43,03	7,42	4,14	5,45	6,11	10,48	7,86	10,26	1,31
0	1,8	1	85	97	00:00:15	67	35,4	0	0	0,6	0,5	0	0	0	0	214,5	12,79	38,83	15,25	8,04	7,87	11,15	10,33	11,64	8,69	0,98
0,71	0,3	0,1	93	96	00:00:00	67	16,3	0	0	0,9	0,5	0,5	0	0	0	154,37	2,71	16,47	14,23	12,55	9,57	8,41	7,11	2,58	0	0,12
0	2,1	1,3	86	97	00:00:20	66	43,8	0	0	0	0,2	0	0	0	0	237,21	43,96	35,26	17,71	15,38	10,99	13,44	16,55	14,22	5,56	0,9
0	0,5	0,3	88	96	00:00:02	48	42,5	0	0	0,7	0,4	0	0	0	0	258,49	10,87	40,78	18,9	14	11,55	14,41	13,46	13,19	4,75	0,67
0	0,9	0,1	92	95	00:00:00	48	48	0	0	0	0	0	0	0	0	304,41	72,95	69,51	17,26	18,3	20,11	18,43	11,03	2,85	0,12	0
0	2,4	1,9	87	97	00:00:16	66	51,9	0	0	0	0,2	0	0	0	0	242,42	62,84	49,26	17,95	13,16	11,25	11,97	12,68	12,44	4,54	0,59
0	0,5	0,4	86	97	00:00:10	63	23,5	0	0	0,5	0,3	0	0	0	0	270,77	17,75	38,31	20,21	14,25	10,88	7,51	6,47	6,6	4,01	1,81
0,56	1,1	0,7	91	96	00:00:00	63	16,9	0	0	0,6	0,4	0,8	0	0	0	139,87	18,06	34,75	8,97	11,71	12,95	10,59	4,98	0,87	0,62	0,24
0	2,7	2,7	86	96	00:00:20	63	30	0	0	0,1	0,3	0	0	0	0	216,91	29,86	34,12	13,68	14,59	17,1	12,99	7,52	1,59	0,45	0,45
0	0,9	0,5	87	97	00:00:07	66	50	0	0	0	0	0	0	0	0	236,66	67,92	58,11	23,84	13,2	11,14	8,4	8,74	3,08	1,37	0,34
0	0,8	0,8	87	96	00:00:09	51	35,8	0	0	0	0	0,2	0	0	0	169,96	36,44	43,38	21,32	16,28	17,31	11,5	10,08	6,46	0,12	0,25
0	0,4	0,4	93	98	00:00:00	72	39,4	0	0	0	0,4	0	0	0	0	219,38	40,73	35,19	15,26	9,71	7,44	8,32	12,23	19,54	12,61	1,63
0,05	4,8	3	88	96	00:00:03	70	51	0	0	0	0,5	0	0	0	0	186,55	49,99	49,29	11,27	11	9,19	15,17	17,96	15,03	5,56	0,97
0,24	8,1	6,4	83	96	00:02:25	59	31,4	0	0	0	0	0,9	0	0	0	135,26	45,15	29,06	21,03	21,26	19,32	13,26	7,65	4,68	0,68	0,22

PW_TIME	SPO2_INDEX	TIME_BELOW_90	TIME_PER_DESAT_DUR	TIME_PER_SYM_DESAT_DUR
27785	26,94	15,25	22,79	9,19
26844	5,23	0	0	0
25920	6,25	0,28	0	0
27711	3,89	0,03	0	0
27566	2,35	0	0	0
27683	0,91	0,05	0	0
27132	3,05	0,03	0	0
27686	17,55	0,2	4,69	0
25590	7,73	0,81	0	0
27947	3,47	0	0	0
27391	13,14	0,43	0	0
27871	5,42	0	3,5	3,33
27570	2,87	0	0	0
29560	2,43	0	0	0
26059	4,55	0,08	2,9	0
27883	3,35	0,28	0	0
24159	2,98	0,05	0	0
27770	13,09	0,31	0	0
16288	16,13	0,01	0	0
23038	2,81	0,58	0	0
19983	0,72	0	0	0
27641	19,27	1,61	18,6	6,44
20954	6,7	3,21	0	0
24160	3,27	0	0	0
26084	19,59	2,53	28,1	3,13
20515	42,46	9,68	38,2	4,34
27559	7,57	0,11	0	0
28806	6,87	0	0	0
27683	20,93	1,61	0	0
24218	36,27	9,83	1,85	0
27543	2,48	0	0	0
27881	5,03	0	0	0
27646	2,86	1,14	0	0
24137	10,14	0,46	0	0
27505	14,26	0,5	9,8	0
22164	7,95	0,41	0	0
16485	5,89	0,23	0	0
21940	10,5	0,18	0	0
27810	1,42	0	0	0
27838	3,75	0,3	0	0
26474	2,17	0,05	0	0
27734	12,72	0	0	0
30074	5,14	0,31	0	0
27784	1,55	0,18	0	0
28891	5,48	0	0	0
15789	12,76	0,21	0	0
20987	2,05	0,06	0	0
27853	7,88	0,06	0	0
28545	2,52	0	0	0
25853	19,77	0,01	3,4	0
31489	21,72	2,51	38,4	0
